# The effect of comprehensive multidisciplinary pulmonary rehabilitation on 5-year survival in COPD: does maintaining a home exercise program improve survival?

**DOI:** 10.55730/1300-0144.5524

**Published:** 2022-07-28

**Authors:** Seher SATAR, Mustafa Engin ŞAHİN, Pınar ERGÜN

**Affiliations:** Department of Chest Diseases, Ankara Atatürk Sanatorium Training and Research Hospital, Health Sciences University, Turkey

**Keywords:** Chronic obstructive pulmonary disease, pulmonary rehabilitation, home exercise program, maintenance, 5-year survival

## Abstract

**Background/aim:**

Chronic Obstructive Pulmonary Disease (COPD) is one of the most common causes of death worldwide. Therefore, optimizing medical therapy in the comprehensive management of the disease, as well as including pulmonary rehabilitation (PR) in the treatment, is essential. The goal of our study was to determine the impact of PR on the survival of COPD patients.

**Materials and methods:**

Between 2007–2015, 509 COPD patients who completed the PR constituted the PR group, while 167 patients who applied but could not complete it after the initial evaluations formed the control group. In the PR group, dyspnea perception, exercise capacity, muscle strength, body composition, quality of life, psychosocial status, and i-BODE scores were assessed at the beginning and end of the program, whereas in the control group, these assessments could only be conducted at the beginning. Also, after PR, our PR participants have prescribed a home exercise program, and they were recalled to the hospital at the 3rd, 6th, 12th, 18th, and 24th months for follow-up visits.

**Results:**

A statistically significant improvement was found in almost all the data (except FEV1/FVC, BORG after exercise, and FFMI) after PR. There was a statistically significant difference in 5-year survival in favor of the PR group (p = 0.006), and in PR patients who accompanied the home exercise program vs. those who did not (p = 0.000). Also the gains in MRC (p = 0.003; OR: 2.20; CI: 1.319–3.682), MEP (p = 0.041; OR: 1.02; CI: 1.001–1.035), and i-BODE (p = 0.006; OR: 0.914; CI: 0.857–0.974) increased the survival.

**Conclusion:**

Apart from incorporating PR into treatment in the comprehensive management of COPD, we demonstrated that maintaining a home exercise program for at least two years following PR increased 5-year survival significantly.

## 1. Introduction

After ischemic heart disease and stroke, Chronic Obstructive Pulmonary Disease (COPD) is one of the leading causes of death, which is also related to high morbidity and disability [1]. Although low physical activity is shown as the most important predictor of mortality in COPD [2]; frequency of acute exacerbations [3], weight loss (especially low fat free mass index- FFMI) [4], comorbidities (especially coronary artery diseases) [5], and i-BODE index [6] are the other predictors of mortality in COPD. So while managing the symptoms and exacerbations of the disease, an important addition to the pharmacological treatment is to provide nonpharmacological approaches including pulmonary rehabilitation (PR) and self-management education.

In this retrospective controlled study, we aimed to compare the 5-year survival results of COPD patients who completed an 8-week comprehensive, multidisciplinary, outpatient, and directly supervised PR program with those who did not, as well as the 5-year survival of the patients who continued a home exercise program for 2 years after the end of PR and came to their scheduled controls, also the factors affecting survival, and finally share our outcomes as it is one of the few controlled studies with a large number of patients.

## 2. Materials and methods

### 2.1. Study population

**Figure 1:**
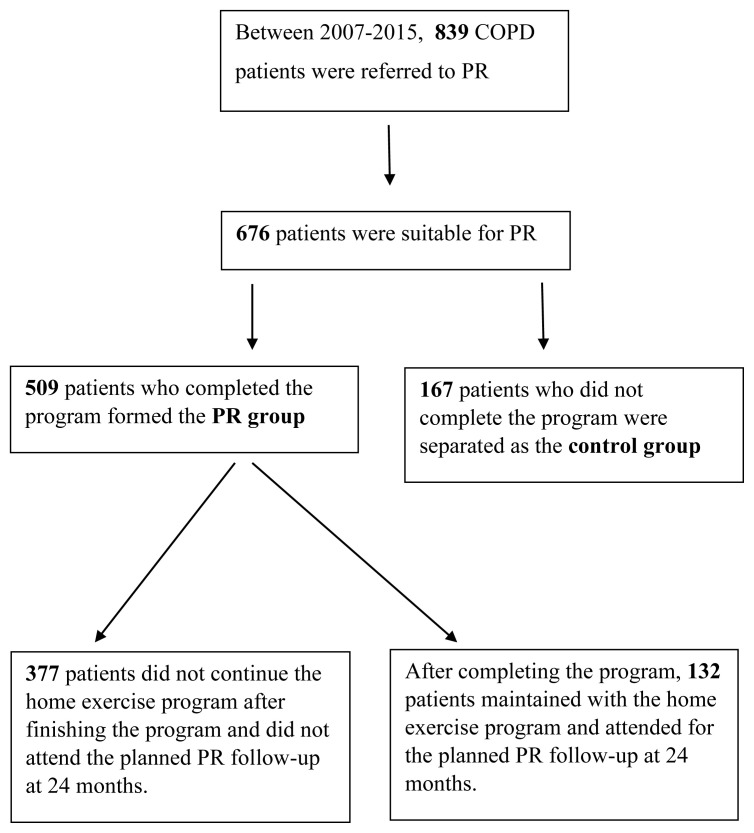
Patient distribution chart.

Between 2007 and 2015, 839 COPD patients were referred to our PR center, however, 163 were considered inappropriate for PR due to conditions such as uncontrolled heart failure, hypertension, cancer, unstable angina pectoris, severe pulmonary embolism or thrombosis, or major neurological diseases. So 676 patients were eligible for the PR program and were included in the study. While 509 patients who completed the program formed the PR group and the remaining 167 patients who did not complete the program for various reasons (active work-life, transportation, or compatibility problems) were separated as the control group. One hundred and thirty-two patients who completed PR maintained the home exercise program prescribed at the end of PR and attended their regularly scheduled sessions for two years, while the remaining 377 patients did not continue their home exercises and did not attend their scheduled sessions (Figure 1). The electronic medical data system at our hospital, as well as the Ministry of Health information system integrated into it, were utilized to screen the 5-year mortality in both the PR and control groups.

### 2.2. Outcome parameters

A spirometry was used to measure pulmonary function [7]. Respiratory muscle strength was assessed using a Micro-RPM (respiratory pressure meter). To examine peripheral muscle strength, hand grip test with hand dynamometer was applied. Incremental shuttle walking test (ISWT) and endurance shuttle walking test (ESWT) were used to assess exercise capacity [8,9]. The St. George’s Respiratory Questionnaire (SGRQ) was used to measure health-related quality of life (HRQL) [10]. The medical research council (MRC) scale was used to measure dyspnea [11]. Body compositions were determined by the bioelectrical impedance method using TANITA (TBF-300A Total Body Composition Analyzer, Tokyo, Japan). Psychological state was determined using Hospital Anxiety and Depression (HAD) scores [12]. Also, i-BODE values were calculated using the body mass index (BMI), forced expiratory volume in one s (FEV1), MRC scale, and ISWT. According to their i-BODE values, patients were split into quartiles, with cut-offs of 0 to 2 points in the first quartile (Q1), 3 to 4 points in the second quartile (Q2), 5 to 6 points in the third quartile (Q3), and 7 to 10 points in the fourth quartile (Q4) [6]. The survival status of all patients was determined by scanning the online database records of the Ministry of Health information system retrospectively.

The primary endpoint of our study was to investigate the effect of 8-weeks of multidisciplinary, comprehensive, outpatient, directly supervised PR, as well as the effect of maintenance of a home exercise program after PR and adhering to a 2-year scheduled follow-up program on 5-year survival in patients with COPD. Our secondary endpoint was to determine which PR gain was associated with the 5-year survival.

### 2.3. Pulmonary rehabilitation

After the evaluation of the patients, an individualized 8-week hospital-based comprehensive PR program for two half days each week was designed. Endurance and resistance training were included in the program. Leg extensions with free weights were performed 2 days a week. Patients with low maximal inspiratory pressure (MIP) values were given inspiratory muscle training (IMT), with an inspiratory load of 30%–50% of MIP. The patients’ heart rates, blood pressure results, and oxygen saturations were monitored during the sessions. If necessary, oxygen supplementation was administered to keep the oxygen level over 90%.

The PR group have also been prescribed a home exercise program, and scheduled follow-up appointments were created at the 3rd, 6th, 12th, 18th, and 24th months to check compliance.

### 2.4. Ethical consideration

We applied to the “Medical Specialization Education Board” in our institution and received approval for our study on 15 October 2020 with decision number 697-7.

### 2.5. Statistical analysis

The Statistical Package for the Social Sciences version 18.0 (SSPS, Chicago, IL, USA) software system was used to conduct statistical analyses. Categorical variables were represented with numbers and percentages (%). The Shapiro-Wilk test was performed to determine the normality of the distribution of the variables. The Chi-square test was employed to examine connections between categorical variables. The McNemar test was used to evaluate categorically dependent data, whereas the Man Whitney U test was used to analyze independent data. Relationships between variables with nonnormal distributions were investigated using Spearman correlation analysis. Relationships between variables were investigated using regression analysis. The 5-year survival was evaluated with the chi-square test. For statistical significance, the p-value was adjusted to be less than 0.05 (p < 0.005).

According to the G-power analysis, the study should have a minimum of 132 controls and 440 study patients, with a confidence interval of 0.90, a level of significance of 0.05, and an effect size of 0.33.

## 3. Results

The mean ages of the PR and control group were 63.95, and 64.22, respectively. Ex-smokers comprised the majority of patients in both groups. All parameters were found to be similar between the two groups except SGRQ, HAD scores, and the total 5-year survival rates. As the primary endpoint of our study, the 5-year survival was significantly higher in the PR group compared to the control group (p = 0.006). Detailed demographic data of both groups are given in Table 1.

When we divided the patients according to i-BODE quartiles, there was no difference between the 5-year survival rates of the patients in both groups (p = 0.084 for Q1, p = 0.125 for Q2, p = 0.399 for Q3, and p = 0.062 for Q4, respectively).

After PR program, statistically significant improvements were detected in nearly all of the patients’ data. The detailed values before and after PR are given in Table 2.

When we consider the effects of PR gains on 5-year survival, only the improvements in MRC, maximal expiratory pressure (MEP), and i-BODE have a statistically significant influence on the survival (p = 0.003, p = 0.007 and p = 0.007, respectively). In the regression analysis of PR group, it was found that gains in MRC (p = 0.003; OR: 2.20; CI: 1.319–3.682), MEP (p = 0.041; OR: 1.02; CI: 1.001–1.035), and i-BODE (p = 0.006; OR: 0.914; CI: 0.857–0.974) increased the survival.

**Figure 2:**
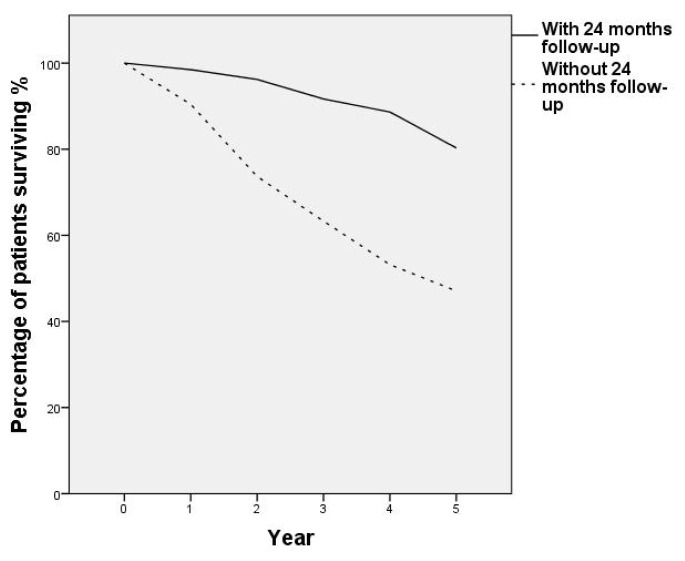
The percentage of 5-year survival by years of patients who maintained the home PR program and attended to their 24-month follow-up versus those who did not maintain the home PR program and did not come for their 24-month follow-up.

Only 132 of the 509 PR patients maintained the home exercise program after finishing the program and came to the hospital for the scheduled PR follow-up at the 24th month, whereas the remaining 377 patients did not. As shown in Table 3 and Figure 2, patients who continued their home exercise programs after PR and participated in PR control after 24 months had a statistically significant positive effect on 5-year survival (p = 0.000). Furthermore, these patients’ BMI, FFMI, SGRQ, and BORG gains remained statistically significant at month 24.

## 4. Discussion

The effect of a multidisciplinary, comprehensive, directly supervised, hospital-based, out-patient PR program on COPD patients’ 5-year survival rates was evaluated in this long-term follow-up study, and when the groups were compared, the PR group had a significantly higher 5-year survival rate. Furthermore, we discovered that patients who continued the home exercise program following PR and attended scheduled hospital follow-ups for up to two years had a significantly better 5-year survival rate than those who did not. We also concluded that gains in MRC, MEP, and i-BODE improve survival.

Dyspnea, presence of comorbidities, BMI, predicted FEV1%, exercise capacity, exacerbations, some biomarkers, inspiratory capacity, and anemia have all been linked to mortality in COPD patients [13].

PR, which is a cost-effective strategy (evidence level 2C), improves exercise tolerance, symptoms, and HRQL in COPD patients (evidence level 1A), as well as reducing hospital stays and healthcare costs (evidence level 2B) [14]. In fact, in the study of Puhan M. et al. [15] pulmonary rehabilitation was shown to be a highly effective and safe intervention to reduce hospitalizations and mortality and improve health-related quality of life in COPD patients even after exacerbation. It is also effective in facilitating smoking cessation, optimizing pharmacotherapy, improving body composition, promoting mental health, managing acute dyspnea, and detecting and managing acute exacerbations [16]. COPD patients have limited physical activity, which is cited as one of the causes of hospital admissions and mortality, due to shortness of breath, muscle weakness, and/or respiratory failure [17].

All demographic data and baseline parameters of our 2 groups were found to be similar except for the quality of life, and psychosocial status which may be related to the inability of the control group to adapt and complete the PR. In the PR group, statistically significant improvements were found in pulmonary function tests, dyspnea perceptions, exercise capacities, respiratory and peripheral muscle strength, body composition, quality of life, psychosocial status, and i-BODE index after PR. Also, the 5-year survival rate of these patients was significantly higher than the control group and we concluded that the benefit of PR in terms of survival was similar to the literature [2,18]. In the publications, respiratory muscle dysfunction has also been identified as a possible predictor of higher use of health resources and mortality in COPD. Our study also showed that the MRC and MEP gains of the patients with 5-year survival were statistically higher than those who did not survive, so the contribution of PR to dyspnea and respiratory muscle strength is associated with survival, and even more objectively, it was found that a 1-unit increase in MRC increased the survival 2.20 times and 1-unit increase in MEP levels increased the survival 1.02 times. According to Houchen-Wolloff L.’s study, better MRC levels, higher baseline ISWT and BMI values, younger age at baseline, and no need for oxygen therapy were associated with higher survival rates [2]. Similar to our study, Decramer M. et al. [19] found that the number of hospital admissions, hospitalization days, and out-patient visits were all related to respiratory and peripheral muscle strength; especially the association was statistically significant for maximal expiratory pressure (PE max). Also, Vilaro J. et al. [20] reported that expiratory muscle weakness is a risk factor for readmission to the hospital because of acute exacerbations which are related to higher mortality rates.

Body composition, airway obstruction, dyspnea perception, and exercise capacity make up the i-BODE index, which is one of the indicators of mortality in COPD. The i-BODE index was found to be an independent and substantial predictor of death in research involving 633 patients who received PR, and each quartile increase in severity in the i-BODE score was significantly related to higher mortality [6]. From another research, the i-BODE index was found to be more directly correlated with survival than the individual components in 674 patients who had 7-week PR [21]. Also, the i-BODE score was significantly lower among the survivors in our study when the data of patients who survived and did not survive for 5 years were compared. However, when our patients were divided into quartiles based on their i-BODE values, there was no statistically significant relationship between percentiles and 5-year survival for both groups. This result appeared to be due to the fact that the number of patients in these two groups was insufficient for statistical analysis, and the PR and control groups’ initial i-BODE values were similar at baseline.

Because low levels of physical activity are linked to mortality, there is an increasing demand for supporting a long-term lifestyle change that includes more activity and less sedentary behavior. According to a recent systematic review and meta-analysis, post-PR patients were followed with supervised maintenance therapy for up to 36 months, and it was discovered that continuous supervised maintenance exercise reduced the number of respiratory hospitalizations and deaths when compared to standard care [22]. Güell MR. et al. [23] revealed that the gain in BODE index and 6MWT continue in the 2-year follow-up after PR in COPD. According to Blervaque L. et al’s study, after a pragmatic maintenance PR program, 144 patients with COPD were followed annually for 5 years and demonstrated significant PR gains in 6MWT and HRQL at 4 years and MRC at 5 years [24]. Furthermore, they discovered that PR patients had a higher 5-year survival rate than the others.

Although there are various studies on the long-term effectiveness of PR in COPD, there are few studies on the benefit of continuing a home exercise program after PR on survival other than the existing gains. In a meta-analysis of the long-term effects of PR with low-frequency maintenance on exercise capacity and health-related quality of life in COPD patients, it was discovered that PR with maintenance programs appears to be more effective than PR without maintenance programs for preserving exercise capacity in the long-term follow-up, but maintenance was not effective on health-related quality of life [25]. The recurrent prescription of home exercise training at 6-month intervals may have contributed to the high 5-year survival rate, according to a study conducted with 33 COPD patients from Turkey [26]. When we compared the PR group patients who continued the home exercise program after completing the PR and came for the scheduled controls, including the 2nd year, to those who did not, we discovered that the 5-year survival in the group who continued the PR program at home and came to the control was better than the other group, and that the gain in BMI, FFMI, SGRQ, and BORG after exercise continued at the 24th month. This demonstrated that patients who finished a directly supervised PR program should continue to exercise at home to preserve their gains and have a higher survival rate.

Our study’s strengths include the fact that it was a randomized controlled trial with a large number of patients, that the program was implemented by a multidisciplinary team in an experienced PR center, and that the patients were followed for an extended period of time with a structured follow-up program. The most significant restriction of our research is that the majority of our data on patients’ compliance with the home maintenance program mainly based on the patient’s statement.

## 5. Conclusion

In this study, we demonstrated that not only completing PR but also continuing a home exercise program and confirming this with directly supervised hospital controls for up to 2 years improves 5-year survival in COPD patients.

## Figures and Tables

**Table 1:** Demographic data of both PR and control groups.

	PR group	Control group	p
Age (years)	63.95 ± 8.60	64.22 ± 9.38	0.757
Sex (m/f) n (%)	446(87.6%)/63(12.4%)	148(88.6%)/9(11.4%)	0.731
Smoking (p/year)	50.11 ± 30.99	50.11 ± 33.24	0.940
LTOT presence n (%)	237 (49.1%)	78 (47.3%)	0.691
FEV1 predicted %	39.18 ± 18.04	38.80 ± 19.81	0.388
FVC predicted %	56.52 ± 18.80	54.36 ± 18.56	0.164
FEV1/FVC	54.80 ± 14.40	54.47 ± 15.52	0.442
MRC score	3.31 ± 0.92	3.41 ± 0.96	0.311
BORG at rest	0.57 ± 0.65	0.56 ± 0.71	0.296
BORG after exercise	3.45 ± 1.10	3.61 ± 1.43	0.162
ISWT (m)	213.72 ± 120.65	214.14 ± 127.43	0.729
ESWT (min.)	7.50 ± 6.88	7.40 ± 6.96	0.757
MIP (cmH2O)	59.88 ± 20.83	60.48 ± 22.26	0.805
MEP (cmH2O)	81.90 ± 39.13	86.77 ± 42.29	0.407
Handgrip (HG) test-R	30.33 ± 8.11	29.57 ± 8.12	0.096
Handgrip (HG) test-L	29.49 ± 7.93	28.45 ± 8.78	0.182
Deltoid strength-R	3.95 ± 0.82	3.81 ± 0.79	0.097
Deltoid strength-L	3.88 ± 0.81	3.77 ± 0.73	0.086
Quadriceps strength-R	4.27 ± 0.73	4.19 ± 0.75	0.299
Quadriceps strength-L	4.20 ± 0.72	4.05 ± 0.72	0.055
BMI (kg/m2)	25.06 ± 6.15	24.87 ± 6.95	0.463
FFMI (kg/m2)	18.83 ± 2.65	18.67 ± 2.86	0.587
SGRQ total score	65.65 ± 17.26	70.54 ± 17.74	**0.001**
HAD-Anxiety scores	9.50 ± 2.34	10.30 ± 2.07	**0.000**
HAD-Depression scores	9.43 ± 2.40	10.16 ± 1.94	**0.000**
i-BODE	4.39 ± 2.15	4.28 ± 2.15	0.727
**Patients living 5 years n (%)**	**305 (60%)**	**80 (47.9%)**	**0.006**

Data are presented as mean ± SD or n (%).

BMI: Body mass index; ESWT: Endurance shuttle walking test; FEV1: Forced expiratory volume in 1 s; FFMI: Fat-free mass index; FVC: Forced vital capacity; HAD: Hospital anxiety depression score; ISWT: Incremental shuttle walking test; L: Left; LTOT: Long term oxygen treatment; m/f: Male/female; MEP: Maximal expiratory pressure; min: Minute; MIP: Maximal inspiratory pressure; MRC: Medical research council; n: Number; p/year: Packet/year; R: Right; SGRQ: St George’s Respiratory Questionnaire.

**Table 2:** Outcome measures before and after the PR program.

Parameters (n = 509)	Before PR	After PR	P-value
FEV1 predicted %	39.18 ± 18.04	40.88 ± 19.25	**0.000**
FVC predicted %	56.51 ± 18.80	58.46 ± 19.07	**0.001**
FEV1/FVC	54.80 ± 14.40	54.86 ± 14.37	0.917
MRC score	3.31 ± 0.92	2.57 ± 0.80	**0.000**
BORG at rest	0.57 ± 0.65	0.35 ± 0.50	**0.000**
BORG after exercise	3.47 ± 1.10	3.40 ± 1.11	0.195
ISWT (m)	213.72 ± 120.65	266.00 ± 125.21	**0.000**
ESWT (min)	7.50 ± 6.88	12.97 ± 7.53	**0.000**
MIP(cmH2O)	59.91 ± 20.87	66.02 ± 23.13	**0.000**
MEP (cmH2O)	81.90 ± 39.12	91.26 ± 43.54	**0.000**
Handgrip test-R	30.33 ± 8.11	32.51 ± 8.24	**0.000**
Handgrip test-L	29.49 ± 7.93	31.75 ± 8.17	**0.000**
Deltoid muscle strength-R	3.95 ± 0.82	4.27 ± 0.77	**0.000**
Deltoid muscle strength-L	3.88 ± 0.80	4.15 ± 0.79	**0.000**
Quadriceps muscle strength-R	4.27 ± 0.72	4.60 ± 0.60	**0.000**
Quadriceps muscle strength-L	4.20 ± 0.72	4.53 ± 0.63	**0.000**
BMI (kg/m2)	25.06 ± 6.15	25.33 ± 5.97	**0.000**
FFMI (kg/m2)	18.83 ± 2.64	18.86 ± 2.49	0.488
SGRQ Score	65.60 ± 17.26	35.12 ± 14.99	**0.000**
HAD-Anxiety	9.50 ± 2.34	6.57 ± 2.55	**0.000**
HAD-Depression	9.43 ± 2.40	6.68 ± 2.91	**0.000**
i-BODE	4.64 ± 2.13	3.42 ± 1.95	**0.000**

Data are presented as mean ± SD or n (%).

BMI: Body mass index; ESWT: Endurance shuttle walking test; FEV1: Forced expiratory volume in 1 s; FFMI: Fat-free mass index; FVC: Forced vital capacity; HAD: Hospital anxiety depression score; ISWT: Incremental shuttle walking test; L: Left; MEP: Maximal expiratory pressure; min: Minute; MIP: Maximal inspiratory pressure; MRC: Medical research council; n: Number; p/year: Packet/year; R: Right; SGRQ: St George’s Respiratory Questionnaire.

**Table 3:** Relationship between 5-year survival in PR group with and without 24-month scheduled follow-up after the program.

PR group patients	Patients with follow-up for 24 months n (%)	Patients without follow-up for 24 months n (%)	P-value
**Survivors**	116 (87.7%)	189 (50.1%)	**0.000**
**Nonsurvivors**	16 (11.9%)	188 (49.9%)	**0.000**

n: Number; PR: Pulmonary rehabilitation
